# Activity of Metabotropic Glutamate Receptor 4 Suppresses Proliferation and Promotes Apoptosis With Inhibition of Gli-1 in Human Glioblastoma Cells

**DOI:** 10.3389/fnins.2018.00320

**Published:** 2018-05-15

**Authors:** Zhichao Zhang, Xiaoyan Zheng, Yan Luan, Yingfei Liu, Xingxing Li, Chongxiao Liu, Haixia Lu, Xinlin Chen, Yong Liu

**Affiliations:** ^1^Institute of Neurobiology, Xi'an Jiaotong University Health Science Center, Xi'an, China; ^2^Department of Human Anatomy, Histology and Embryology, Xi'an Jiaotong University Health Science Center, Xi'an, China; ^3^Department of Neurosurgery, Second Affiliated Hospital, Xi'an Jiaotong University, Xi'an, China

**Keywords:** glioblastoma multiforme, metabotropic glutamate receptor 4, Gli-1, proliferation, apoptosis, LN229 cells

## Abstract

Glioblastoma multiforme (GBM) is the most lethal glioma variant in the adult brain and among the deadliest of human cancers. Increasing evidence has shown that metabotropic glutamate receptor subtype 4 (mGluR4) expression may play roles in regulating the growth of neural stem cells as well as several cancer cell lines. Here, we investigated the effects of mGluR4 on the growth and apoptosis of the LN229 GBM cell line. Involvement of Gli-1, one of the key transcription factors in the sonic Hedgehog (SHH) signaling pathway, was further explored. In this study, mGluR4 was activated using selective agonist VU0155041; and gene-targeted siRNAs were used to generate loss of function of mGluR4 and Gli-1 in LN229 cells. The results demonstrated that LN229 cells expressed mGluR4 and the agonist VU0155041 decreased cell viability in a dose- and time-dependent manner. Activation of mGluR4 inhibited cyclin D1 expression, activated pro-caspase-8/9/3, and disrupted the balance of Bcl-2/Bax expression, which indicated cell cycle arrest and apoptosis of LN229 cells, respectively. Furthermore, Gli-1 expression was reduced by mGluR4 activation in LN229 cells, and downregulation of Gli-1 expression by gene-targeted siRNA resulted in both inhibition of cell proliferation and promotion of apoptosis. Moreover, VU0155041 treatment substantially blocked SHH-induced cyclin D1 expression and cell proliferation, while increasing TUNEL-positive cells and the activation of apoptosis-related proteins. We concluded that activation of mGluR4 expressed in LN229 cells could inhibit GBM cell growth by decreasing cell proliferation and promoting apoptosis. Further suppression of intracellular Gli-1 expression might be involved in the action of mGluR4 on cancer cells. Our study suggested a novel role of mGluR4, which might serve as a potential drug target for control of GBM cell growth.

## Introduction

Glioblastoma multiforme (GBM), known as glioblastoma and classified as grade IV astrocytoma, is the most common and lethal tumor originating from the adult brain (Tanaka et al., 2013). GBM is characterized by rapid progression and intensive migration and is highly infiltrative to the surrounding brain tissue (De Witt Hamer, 2010). The standard therapy for GBM includes surgical resection followed by radiation in combination with chemotherapy (Stupp et al., 2005). However, this type of tumor is resistant to both radiotherapy and most chemotherapeutic agents. Even following maximum treatment, the cancer usually recurs, 5-year survival is rare (>3–5%), and life expectancy following diagnosis is scarce (12–15 months) (Schwartzbaum et al., 2006; Wen and Kesari, 2008). Therefore, it is crucial to explore alternative methods to improve existing therapies.

Metabotropic glutamate receptors (mGluRs) are usually expressed in mature neurons and neuroglia in the central nervous system (CNS), activated by the excitatory neurotransmitter glutamate, and play an essential role in regulation of synaptic transmission. The mGluRs comprise eight subtypes of a family of G-protein-coupled receptors. Based on their pharmacological properties and signaling pathways, mGluRs can be classified into three groups in which group I (mGluR1 and mGluR5) positively coupled to phosphoinositide hydrolysis, group II (mGluR2, and mGluR3) and group III (mGluR4, mGluR6, mGluR7, and mGluR8) are both negatively coupled to adenylate cyclase (Rosemond et al., 2002; Byrnes et al., 2009). Recently, researchers provided evidence that mGluR subtypes are expressed in undifferentiated brain cells and several types of cancer cells, and may serve as a regulator for survival, proliferation, and differentiation of these cell types (Pollock et al., 2003; Canudas et al., 2004; Melchiorri et al., 2007; Nicoletti et al., 2007; Spampinato et al., 2014). Specifically, mGluR4 present in neural stem cells reduces cell proliferation and promotes neuronal differentiation (Canudas et al., 2004; Saxe et al., 2007; Nakamichi et al., 2008; Zhang et al., 2015). Similarly, the receptor is markedly expressed when embryonic stem cells differentiate into neural precursors (Cappuccio et al., 2006). Interestingly, the existence of mGluR4 has been documented in medulloblastoma arising from the cerebellum of children (Iacovelli et al., 2006) and several non-neural malignant tumors, such as colorectal carcinoma (Yoo et al., 2004; Chang et al., 2005) and osteosarcoma (Yang et al., 2014). There are conflicting reports in published literature regarding the function of the receptor in different tumor cells. Expression of mGluR4 may be connected with 5-fluorouracil resistance in a human colon cancer cell line (Yoo et al., 2004), and poor prognosis in osteosarcoma (Yang et al., 2014). However, activation of mGluR4 in medulloblastoma cells reduces the ectopic tumor growth in nude mice (Iacovelli et al., 2006). Nonetheless, mGluR4 may implicate a novel target to control the growth of tumors, especially brain tumors. In this regard, we hypothesize that mGluR4 is expressed in and plays a role in regulating the growth of GBM cells.

Glioma-associated oncogene homolog 1 (Gli-1), originally identified in human glioma cells (Kinzler et al., 1987), is a zinc finger-containing transcription factor and the main nuclear mediator of the Sonic Hedgehog (SHH) signaling pathway (Dahmane et al., 2001; Infante et al., 2015) that regulate cell proliferation and differentiation during CNS development (Briscoe and Therond, 2013). However, Gli-1 is highly expressed in diverse cancer cells and critically involved in the tumorigenesis and cancer growth, including GBM (Sanai et al., 2005; Wang et al., 2010). Although it is currently unknown that if mGluR4 can regulate the expression and function of Gli-1 in tumor cells, a study in rat cerebellar granular cell precursors indicated mGluR4 activation inhibited the expression of Gli-1 (Canudas et al., 2004), which may suggest a potential intracellular molecule of mGluR4 in regulation of tumor growth.

In this regard, we hypothesize that mGluR4 is expressed in GBM cells; activity of mGluR4 may regulate the growth of the tumor cells; and Gli-1 transcript factor may be an intracellular target of mGluR4. In the present study, LN299 cells, one of the GBM cell lines, were used to investigate the role of mGluR4 in controlling the growth and apoptosis of GBM cells. We utilized the drug, cis-2-[(3, 5-dichlorophenyl) aminocarbonyl] cyclohexanecarboxylic acid (VU0155041, VU), which is a new subtype-selective agonist of mGluR4 (Niswender et al., 2008; Zhang et al., 2015) to activate mGluR4. Effects of mGluR4 on the proliferation and apoptosis of the malignant tumor cells were first examined. Moreover, possible involvement of Gli-1 were further determined in LN229 cells to explore the underlying mechanisms of mGluR4 regulation of GBM cell growth.

## Materials and methods

### LN229 cell culture and treatment

The LN229 human glioblastoma cell line was purchased from the American Type Culture Collection (ATCC, USA). Cells were plated into T25 flasks and maintained in Dulbecco's Modified Eagle's Medium (DMEM) supplemented with 10% fetal bovine serum (FBS), 100 U/mL penicillin and 100 μg/mL streptomycin (all from Gibco, USA). Cells were incubated at 37°C in an incubator (SANYO, Japan) with humidified 5% CO_2_/95% air. Cells were passaged every 3–5 days, and cryopreserved with liquid nitrogen tank when necessary. The drug VU0155041 sodium was obtained from Tocris (UK) and dissolved in sterile 0.9% normal saline (NS). The recombinant human sonic Hedgehog protein (shh) was purchased from R&D Systems (USA) and reconstituted in sterile 0.01 M phosphate buffered saline (PBS) containing 0.1% bovine serum albumin (BSA).

### Immunofluorescence staining

LN229 cells were plated onto coverslips and fixed with 4% paraformaldehyde (PFA) at room temperature (RT) for 20 min followed by washing three times in PBS. Before blocking with BSA (5% in PBS) for 1 h, the cells were permeabilized in 0.1% Triton X-100 for 10 min. The primary rabbit anti-mGluR4 polyclonal antibody (1:200, Abcam, UK) was added and incubated overnight at 4°C. The cells were then incubated with Alexa Fluor 594 conjugated anti-rabbit IgG (1:800, Invitrogen, USA) at RT for 2 h. Cell nuclei were counterstained using 4′,6-diamedino-2-phenylindole (DAPI, 1 μg/mL). Coverslips were mounted on slides by antifade mounting medium (Molecular probes, USA). The immunostaining negative control was generated by replacing the primary antibody with 5% BSA, and neural stem cells, which show functional expression of mGluR4 (Saxe et al., 2007; Zhang et al., 2016), were used as a positive control. Images were acquired using a fluorescence microscope equipped with a digital camera (BX51 + DP71, Olympus, Japan) and analyzed with ImageJ software (NIH, USA).

### siRNA transfection

All siRNA duplexes were designed and synthesized by Genepharma Corporation (China). The siRNA sequences targeting Gli-1 and mGluR4 were as follows: siGli-1-1, 5′-GGCUCAGCUUGUGUGUAAUTT-3′; siGli-1-2, 5′-CUCCACAGGCAUACAGGAUTT-3′; simGluR4-1, 5′-GCAUGUCACCAUAAUUUGCTT-3′; simGluR4-2, 5′-GGUCAUCGGCUCAUGGACATT-3′; A non-specific siRNA (siNC, 5′-CGTACGCGGAATACTTCGATT-3′) was used as a negative control for the siRNA transfection experiments. siRNA transfection was performed as previously described with minor modifications (Zhang et al., 2015). In brief, LN229 cells were cultured in 24-well plates. Before siRNA transfection, cells were rinsed three times using pre-warmed Opti-MEM medium (Invitrogen). The cells were incubated with 100 nM of siRNA duplexes in the presence of Lipofectamine 2000 (Invitrogen, USA) and Opti-MEM for 6 h at 37°C. Then, the medium was replaced, and the cells were cultured for 24 h before further processing. siRNA transfection efficiency was evaluated by a fluorescence-labeled non-specific siRNA. Depletion of mGluR4 and Gli-1 expression was confirmed by western blot analysis at 24 h post-transfection.

### Cell viability assay

Cell viability was assessed using a 3-(4, 5-dimethylthiazol-2-yl)- 2,5-diphenyl tetrazolium bromide (MTT)-based assay. LN229 cells were cultured in 96-well plates for 24 h prior to the experiments. At the indicated time points, 20 μL of MTT (5 mg/mL, Sigma-Aldrich, USA) was added to each well and incubated for 2 h at 37°C. Then, the medium was aspirated and the resulting insoluble purple crystals were dissolved using 150 μL dimethyl sulfoxide (DMSO, Sigma-Aldrich). Absorbance at 490 nm was read using a multi-microplate spectrophotometer (BioTek, USA). Triplicate wells were subjected to each treatment and at least three independent experiments were performed. The assay data were presented as the percentage of A490 of each treatment to that of the control cells.

### Fluorescence-activated cell sorting (FACS) assay

Cell cycle and apoptosis were analyzed by FACS assay. LN229 cells were plated into T25 flasks and subjected to treatments. At the predetermined time points, the adherent cells were collected by trypsin digestion. Cell cycle was measured as previously described (Chen et al., 2010). Briefly, cells were fixed in ice-cold 75% ethanol overnight at 4°C. After three washes in PBS, the cells were incubated with 100 μg/mL propidium iodide (PI) containing 100 μg/mL RNase A for 30 min at 37°C in the dark. Then, FACS assay was performed using a FACS Calibur (BD Biosciences, USA) with excitation at λ488 nm and emission at λ630 nm. For each sample, 20 000 cells were detected. Data were collected and analyzed using FACSort CellQuest software and Modfit LT software (both from BD Biosciences), respectively. The changes in cell cycle distribution were demonstrated by the proliferation index. The following formula was used: proliferation index = [(S+G2/M)/(G0/G1+S+G2/M)] × 100%. Apoptosis analysis was conducted with Annexin V-FITC and PI staining using a Cell Apoptosis Kit (Invitrogen) according to the manufacturer's instructions. In brief, cells were suspended in 100 μL PBS, mixed with 5 μL of Annexin V-FITC and 1 μL of PI (100 μg/mL) and incubated at RT for 20 min. Then 400 μL of 1x Annexin-binding buffer was added, and the cells were immediately placed on ice. Cell apoptosis was analyzed with a BD FACS Calibur. The percentage of early apoptotic cells (Annexin V^+^/PI^−^, lower right quadrant) plus later apoptotic cells (Annexin V^+^/PI^+^, upper right quadrant) was calculated. At least three independent experiments were performed in each assay.

### TUNEL staining

Cell apoptosis was detected using a terminal-deoxynucleotidyl transferase-mediated dUTP nick end labeling (TUNEL) assay kit (Roche Diagnostics, USA) according to the manufacturer's instructions. In brief, LN229 cells were plated onto coverslips. After treatments, the cells were fixed with 4% PFA for 20 min, followed by permeabilization using 0.1% Triton X-100 in 0.1% sodium citrate buffer for 2 min on ice. The cells were further stained with 50 μL TUNEL reaction mixture for 1 h at 37°C and extensively rinsed. Counterstaining was performed with DAPI (1 μg/mL) and coverslips were mounted onto the glass slides. Images were collected using a fluorescence microscope with a 40 × objective (BX51 + DP71, Olympus) and processed with ImageJ software. For cell death analysis, TUNEL stained cells in 10 randomly selected fields of each sample were counted, and triplicate samples were used for each treated group. At least three independent experiments were carried out. The percentage of TUNEL-stained cells among the total cells (DAPI-stained cells) was determined to evaluate cell death.

### Western blot analysis

LN229 cells were cultured on 6-well plates and treated. Cells were harvested with RIPA lysis buffer (Pierce, USA) containing anti-protease cocktail (Roche, USA) and cleared by centrifugation at 4°C. Protein concentrations were measured by BCA reagents (Pierce). Twenty to forty micrograms of each sample was subjected to 8 or 12% SDS-PAGE and transferred to polyvinylidene fluoride membranes (Bio-Rad, USA). Membranes were blocked with 5% non-fat milk for 1 h, followed by incubation with specific primary antibodies. The primary antibodies and dilutions used in the experiments were as follows: rabbit anti-mGluR4 (1:1,000, Abcam); rabbit anti-Gli-1 polyclonal (1:1,000, Cell Signaling Technology); rabbit anti-caspase 3 polyclonal (1:1,000, Cell Signaling Technology); rabbit anti-caspase 8 polyclonal (1:1,000, Cell Signaling Technology); rabbit anti-caspase 9 polyclonal (1:1,000, Cell Signaling Technology); mouse anti-Bcl-2 monoclonal (1:1,000, Millipore); mouse anti-Bax monoclonal (1:1,000, Millipore); mouse anti-cyclin D1 monoclonal (1:1,000, Cell Signaling Technology); mouse anti-β-actin monoclonal (1:10,000, Sigma-Aldrich). Then, membranes were incubated with horseradish peroxidase-conjugated anti-rabbit or anti-mouse secondary antibodies (1:100,000, Sigma-Aldrich) for 1 h, and processed for visualization with enhanced chemiluminescent substrate (Pierce) followed by development on X-ray films (Fuji, Japan). Immunoblot bands were imaged by a gel imaging system (G: Box, Syngene, UK) and analysis of band intensity was performed with ImageJ software. The expression levels of investigated proteins were determined and normalized to the internal control, β-actin. All western blot data represent at least three separate experiments.

### Statistical analysis

Data are shown as the mean ± SD of at least three independent experiments (*n* = 3–6, which always refers to independent experiments). Each experiment was run in triplicate or quadruplicate. Statistical comparisons were carried out by one-way ANOVA followed by Tukey's test with SPSS software (Version 23.0). *P* < 0.05 was considered as the standard for statistical significance.

## Results

### Activation of mGluR4 reduces cell viability of LN229 cells in a dose- and time-dependent manner

**Figure 1:**
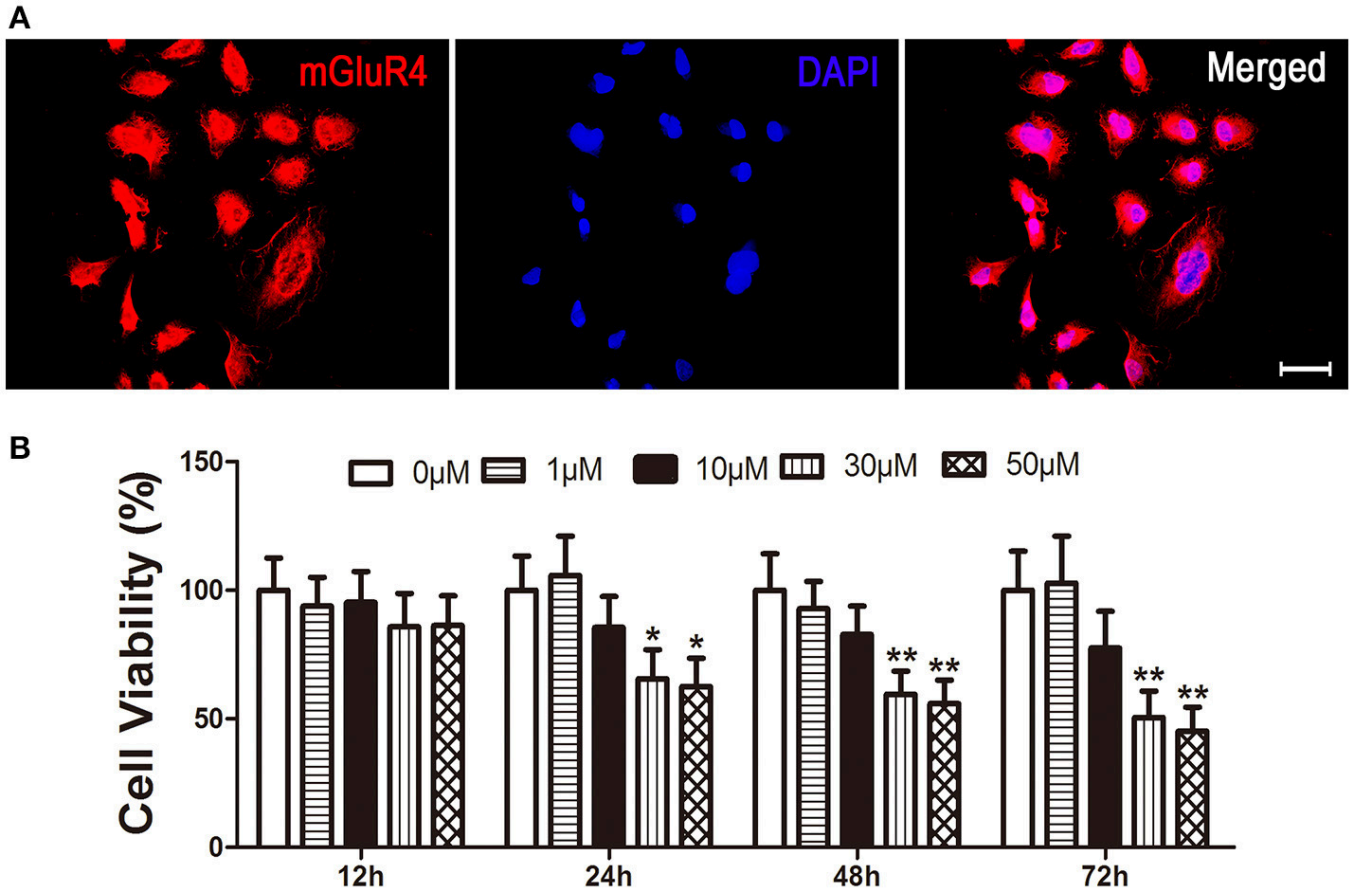
Activation of mGluR4 reduces viability of LN229 cells. **(A)** mGluR4 expression in LN229 cells was determined by immunofluorescence (red), and nuclei were counter-stained with 4′,6-diamedino-2-phenylindole (DAPI, blue). Scale bar = 50 μm. **(B)** LN229 cells were exposed to different concentrations of VU0155041 (0, 1, 10, 30, and 50 μM) for different durations (12, 24, 48, and 72 h). Then, the time- and dose-dependent effects of mGluR4 activation on cell viability were evaluated using 3-(4, 5-dimethylthiazol-2-yl)-2, 5-diphenyl tetrazolium bromide (MTT) assay. Cell viability is presented as a percentage of the control, and each value represents the mean ± SD of three independent experiments. ^*^P < 0.05, ^**^P < 0.01 vs. control groups, respectively.

Expression of mGluR4 in LN229 cells was determined by a specific primary antibody using immunofluorescence staining. The results showed that 95 ± 5% of the LN229 cells expressed mGluR4 (Figure 1A, Figure S1). To identify the effect of mGluR4 activation on cell viability, LN229 cells were treated with serial concentrations of a specific mGluR4 agonist, VU (1, 10, 30, and 50 μM) for 12, 24, 48, and 72 h. MTT assay showed that VU treatments decreased viability of LN229 cells in a time- and dose-dependent manner. Treatments with 30 or 50 μM of VU induced significant reduction of cell viability at 24, 48, and 72 h, compared that of controls (Figure 1B). Because there was no significant difference in cell viability between 30 and 50 μM VU treatments, the lower dose of 30 μM VU was selected for further experiments.

### Activation of mGluR4 inhibits cyclin D1 expression in LN229 cells

**Figure 2:**
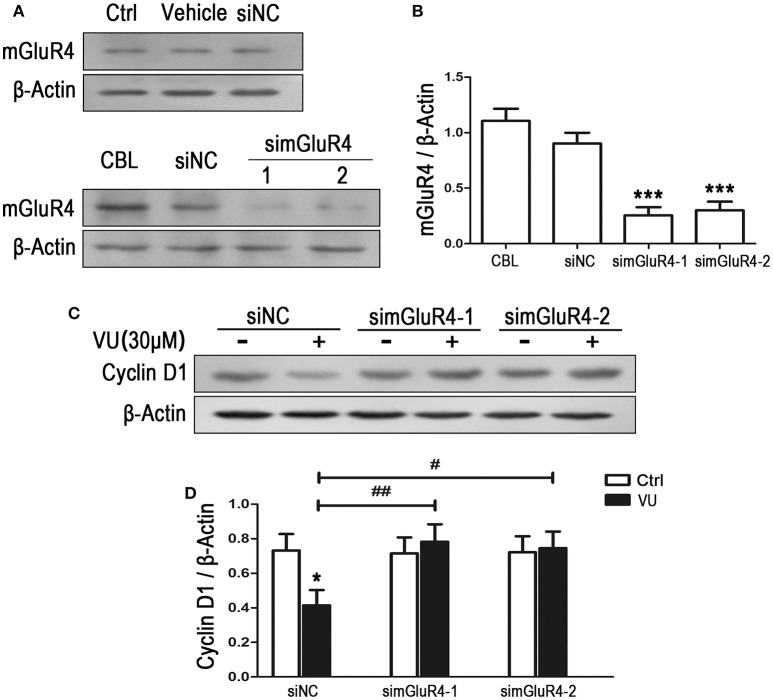
mGluR4 activation inhibits the expression of cyclin D1 in LN229 cells. **(A)** LN229 cells were transfected with vehicle only, non-specific siRNA (siNC), and two mGluR4-targeted siRNAs (simGluR4-1 and simGluR4-2) using Lipofectamine 2000. mGluR4 protein levels were examined by western blot (WB). Samples isolated from cerebellar tissue (CBL) were used as a method control. **(B)** WB bands were quantified to generate the ratio of mGluR4 to β-actin for estimation of the downregulation of mGluR4 gene expression. ^***^P < 0.001 vs. siNC-transfected cells. **(C)** The transfected LN229 cells were treated with the vehicle (Ctrl) or 30 μM of VU0155041 (VU) for 24 h. Then, the expression of cyclin D1 were determined using WB. **(D)** Quantitative analysis was performed to generate the ratio of cyclin D1 to β-actin, and each value represents the mean ± SD of at least three independent experiments. ^*^P < 0.05 vs. Ctrl group; ^#^*P* < 0.05, ^##^*P* < 0.01 vs. VU0155041 group.

To observe the effect of mGluR4 on proliferation of LN229 cells, mGluR4 gene expression was downregulated using a small interfering RNA technique. Transfection efficiency was determined using a fluorescence-labeled non-specific control siRNA. Western blot analysis revealed that mGluR4 protein expression in LN229 cells was effectively reduced by transfection with gene-targeted siRNAs (simGluR4-1 and simGluR4-2), compared with that following siNC transfection, while transfection with Lipofectamine 2000 only (vehicle) and siNC had no obvious influence on mGluR4 expression, compared with that of non-transfected cells (Figures 2A,B). High expression levels of mGluR4 were found in cerebellar tissue, which was used as a positive control (Figures 2A,B).

Utilizing the mGluR4-downregulated LN229 cells, we examined the influence of VU on the expression of cyclin D1, one of the key regulators of the cell cycle. In the siNC transfected LN229 cells, cyclin D1 was significantly decreased after treatment with 30 μM of VU for 24 h, while VU had no significant influence on cyclin D1 expression in the LN229 cells transfected with mGluR4-targeted siRNAs (Figures 2C,D). Furthermore, transfection with mGluR4-targeted siRNAs rescued the reduction in cyclin D1 induced by VU, compared with that of the siNC-transfected group (Figures 2C,D). These data suggested that mGluR4 activation inhibit the expression of Cyclin D1, which may result in cell cycle arrest and decrease of LN229 cell proliferation.

### Effects of mGluR4 on expression of apoptosis-related proteins in LN229 cells

**Figure 3:**
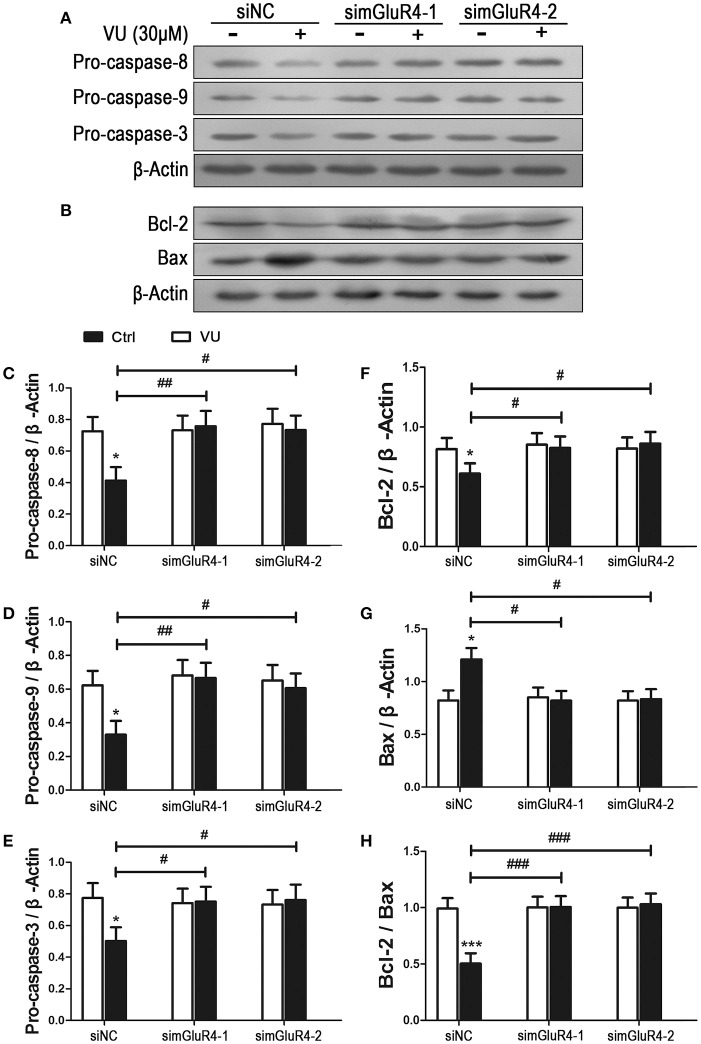
Effects of mGluR4 activation on the expression of apoptosis-related proteins in LN229 cells. **(A,B)** Cultured LN229 cells were transfected with non-specific siRNA (siNC) and two mGluR4-specific siRNAs (simGluR4-1 and simGluR4-2) for 24 h, followed by treatment with the vehicle (Ctrl) or 30 μM of VU0155041 for 24 h. Then, the differential expression of pro-caspase-8/9/3 **(A)** and Bcl-2/Bax **(B)** was determined by western blot (WB) analysis. **(C–H)** WB bands were quantified to generate the ratios of pro-caspase-8 **(C)**, 9 **(D)**, 3 **(E)**, Bcl-2 **(F)**, Bax **(G)** to β-actin and the ratio of Bcl-2 to Bax **(H)**, each statistical value represents the mean ± SD of at least three independent experiments. ^*^P < 0.05, ^***^P < 0.001 vs. Ctrl group; ^#^*P* < 0.05, ^##^*P* < 0.01, ^###^*P* < 0.001 vs. VU0155041 group.

We further determined the effect of mGluR4 activation on LN229 cell apoptosis. Expression levels of pro-apoptotic precursors, pro-caspase-8/9/3, and the anti-apoptotic/apoptotic protein pair Bcl-2/Bax were investigated in LN229 cells by western blot analysis. Participation of these potential intracellular moderators in cellular apoptosis was previously estimated by other investigators by determining their intracellular levels (Elmore, 2007; Ouyang et al., 2012; Zhang et al., 2015). Expression of mGluR4 in LN229 cells was depleted following transfection with mGluR4-specific siRNAs (simGluR4-1 and simGluR4-2), and treatment with the vehicle or 30 μM of VU for 24 h. In siNC-transfected cells, VU treatment led to a significant decrease in pro-caspase-8/9/3 levels. However, in the LN229 cells transfected with mGluR4-specific siRNAs, the levels of these proteins were not markedly influenced by VU treatment. Compared with that of the siNC-transfected cells, the inhibitory action of VU on pro-caspase-8/9/3 expression was significantly attenuated by mGluR4 knockdown (Figures 3A,C–E, Figure S2).

We also assessed the effect of the mGluR4 agonist on expression of the anti-apoptotic/apoptotic protein pair Bcl-2/Bax. Western blot analysis revealed that VU treatment decreased the level of anti-apoptotic protein Bcl-2 and increased the level of apoptotic protein Bax. Hence, the ratio of Bcl-2/Bax was significantly reduced, as compared with that in the control. However, the effect of VU on Bcl-2/Bax expression was markedly prevented by mGluR4-specific siRNA transfection (Figures 3B,F–H).

**Figure 4:**
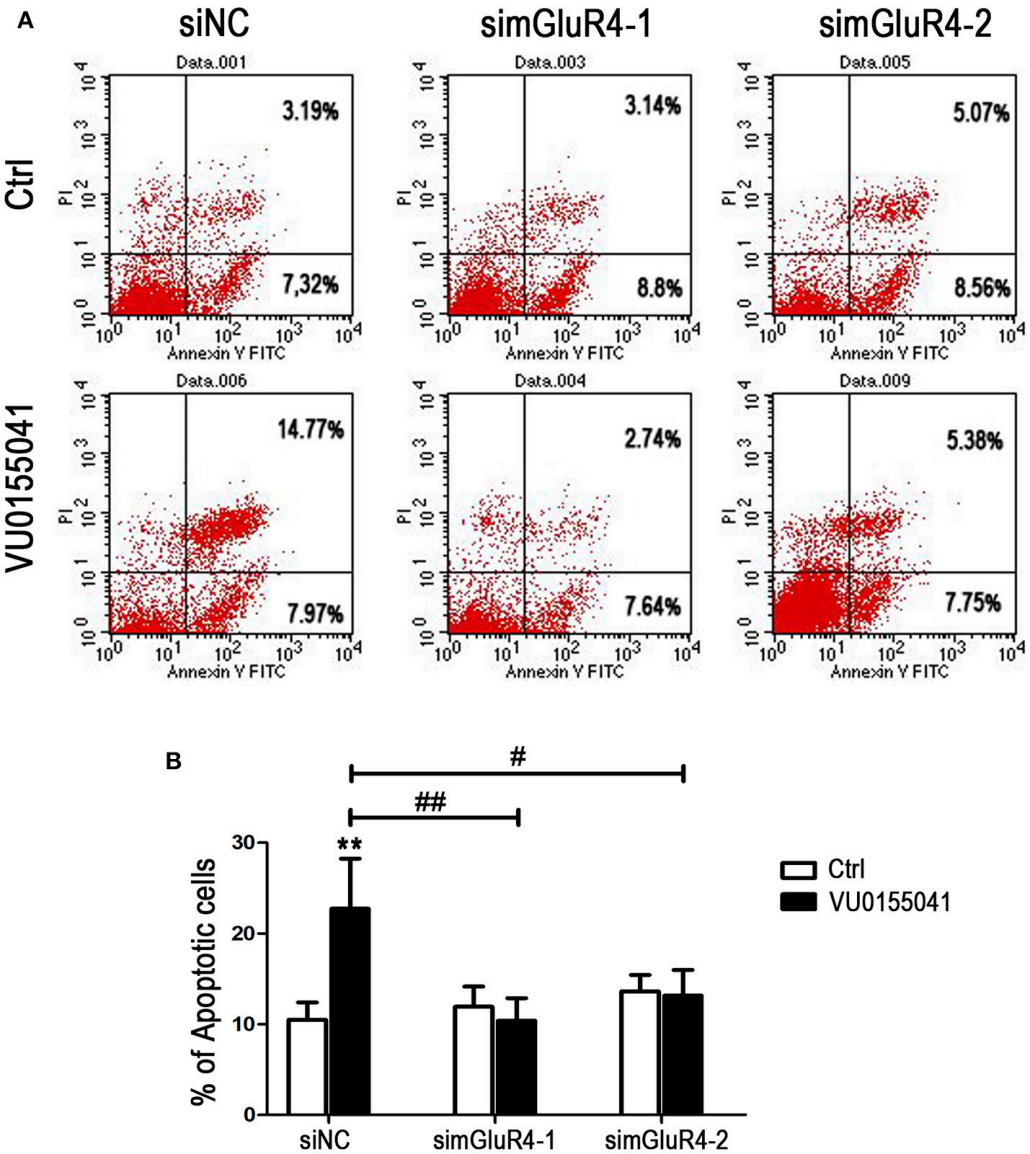
Effects of mGluR4 activation on apoptosis of LN229 cells. **(A)** LN229 cells were transfected with non-specific siRNA (siNC) and two mGluR4-specific siRNA (simGluR4-1 and simGluR4-2) for 24 h, followed by treatment with the vehicle (Ctrl) or 30 μM of VU0155041 for 24 h. Cells were harvested and subjected to Annexin V-FITC and PI staining followed by FACS assay. The representative FACS plots are provided, demonstrating the percentages of early apoptotic cells (Annexin V^+^/PI^−^, lower right quadrant) and later apoptotic cells (Annexin V^+^/PI^+^, upper right quadrant). **(B)** Statistical quantification of cell apoptosis is illustrated. The number of apoptotic cells in each group is provided with the percentage of early apoptotic cells plus later apoptotic cells; statistical values represent the mean ± SD of at least three independent experiments. ^**^P < 0.01 vs. Ctrl group; ^#^*P* < 0.05, ^##^*P* < 0.01 vs. VU0155041 group.

LN229 cell apoptosis was further analyzed using Annexin V-FITC and PI staining followed by FACS assay. In siNC transfected cells, agonist VU significantly induced apoptotic cell death, compared with that of the control. However, after transfection with mGluR4-specific siRNAs, the cell apoptosis induced by VU was abated, as compared with that of untreated counterparts. By comparison with that of the siNC-transfected cells, the enhancement of cell apoptosis by VU was significantly attenuated by mGluR4 downregulation (Figures 4A,B). These results suggested that the effect of mGluR4 activation on apoptosis-related proteins tended to induce the apoptosis of LN229 cells.

### Activation of mGluR4 suppresses expression of Gli-1 in LN229 cells

**Figure 5:**
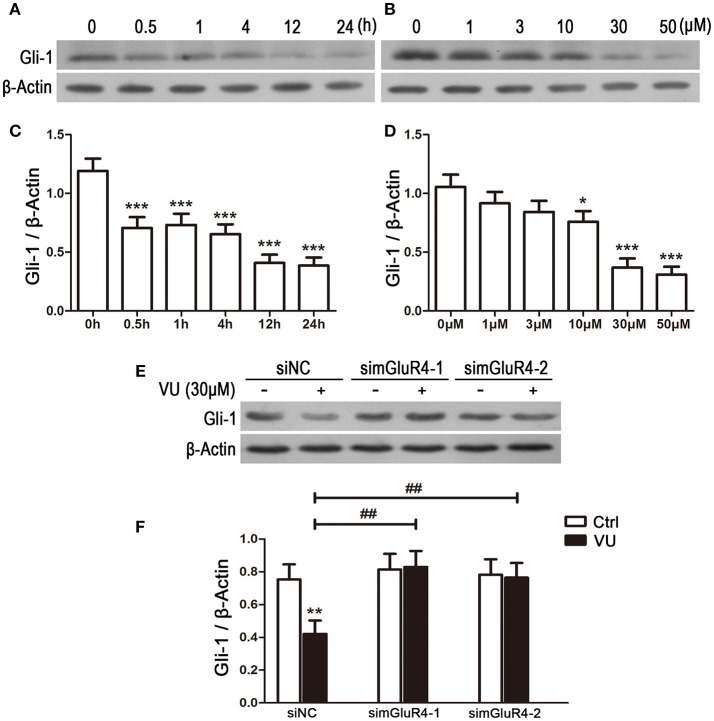
Activation of mGluR4 suppresses the expression of Gli-1 in LN229 cells. **(A,C)** LN229 cells were treated with 30 μM of VU0155041 (VU) for different durations (0, 0.5, 1, 4, 12, and 24 h). Representative western blot (WB) bands **(A)** and quantitative analysis **(C)** are shown to illustrate the time-dependent decrease in Gli-1 expression. ^***^P < 0.001 vs. control. **(B,D)** LN229 cells were treated with serial concentrations of VU (1, 3, 10, 30, and 50 μM) for 24 h. Representative WB bands **(B)** and quantitative analysis **(D)** are presented to illustrate the dose-dependent decrease of Gli-1 expression. ^*^P < 0.05, ^***^P < 0.001 vs. control. **(E,F)** LN229 cells were transfected with non-specific siRNA (siNC) and two mGluR4 specific siRNAs (simGluR4-1 and simGluR4-2), followed by treatment with the vehicle (Ctrl) or 30 μM of VU for 24 h. Then, the expression of Gli-1 was determined by WB **(E)**. WB bands were quantified to generate the ratio of Gli-1 to β-actin **(F)**, and each value represents the mean ± SD of three independent experiments. ^**^P <0.01 vs. Ctrl; ^##^*P* < 0.01 vs. VU group.

To explore the potential link between mGluR4 and regulation of Gli-1 level in LN229 cells, the cells were treated with 30 μM of VU for serial durations (0.5, 1, 4, 12, and 24 h) or with serial concentrations of VU (1, 3, 10, 30, and 50 μM) for 24 h. Then, the expression levels of Gli-1 protein were determined using western blot analysis. As shown in Figure 5, expression of Gli-1 was significantly downregulated by mGluR4 activation, and the agonist VU functioned in a time-dependent manner (Figures 5A,C). Moreover, Gli-1 expression was inhibited by VU in a dose-dependent manner; 10, 30, and 50 μM of VU significantly suppressed Gli-1 expression, while 1 and 3 μM did not (Figures 5B,D).

We further confirmed the inhibitory role of mGluR4 in Gli-1 expression in LN229 cells transfected with mGluR4-targeted siRNA. In the siNC-transfected LN229 cells, Gli-1 expression was decreased by VU treatment, while VU had no significant influence on Gli-1 level in the cells transfected with mGluR4-targeted siRNA. Compared with that of siNC-transfected cells, mGluR4 downregulation significantly attenuated the contribution of VU to repression of Gli-1 expression in LN229 cells (Figures 5E,F). These data suggested that activation of mGluR4 inhibits the expression of Gli-1 and thereby may block the activation of the SHH pathway.

### Effect of Gli-1 downregulation on proliferation and apoptosis of LN229 cells

**Figure 6:**
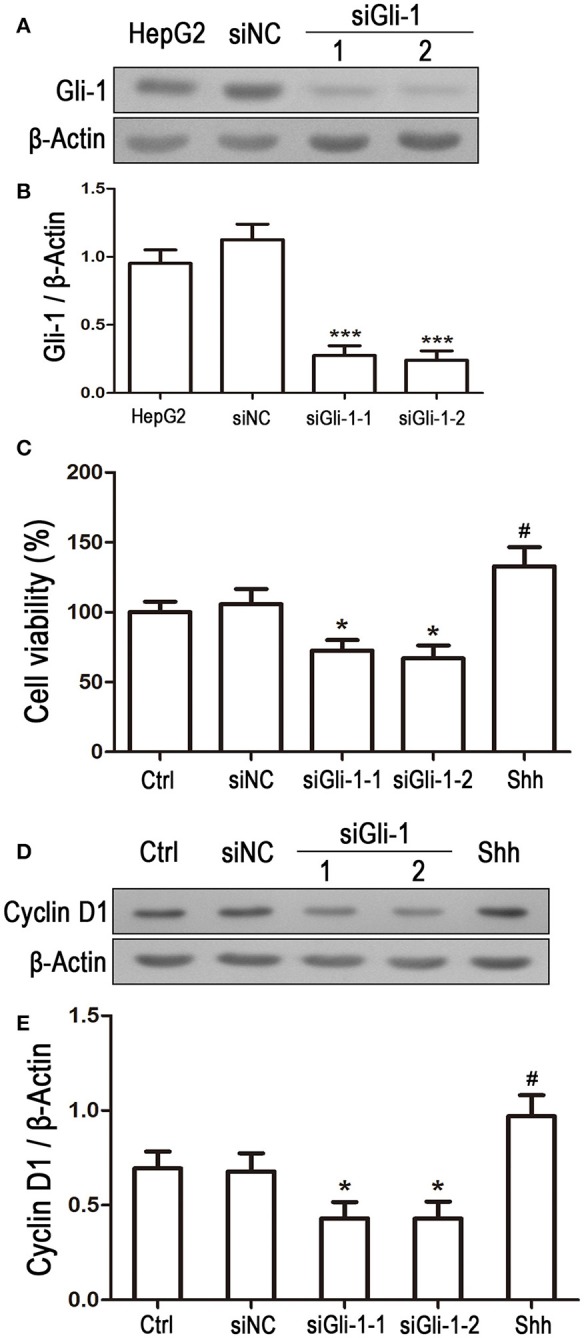
Effect of Gli-1 knockdown on viability and cyclin D1 expression of LN229 cells. **(A)** LN229 cells were transfected with non-specific siRNA (siNC) and two Gli-1-specific siRNAs (siGli-1-1 and siGli-1-2). Gli-1 protein levels were examined using western blot (WB). A sample isolated from the HepG2 cell line was used as a method control. **(B)** WB bands were quantified to generate the ratio of Gli-1 to β-actin for estimation of the knockdown of Gli-1 gene expression. ^***^P < 0.001 vs. siNC-transfected cells. **(C)** Viability of transfected cells was assessed with MTT assay, and shh protein (5 μg/mL), one of the SHH ligands, was used as a positive control. ^*^P < 0.05 vs. siNC group; ^#^*P* < 0.05 vs. Ctrl group. **(D)** Representative WB images illustrate the protein expression of cyclin D1 in siRNA-transfected LN229 cells. Shh protein (5 μg/mL) was added as a positive control. **(E)** Quantitative analysis was performed to generate the ratio of Cyclin D1 to β-actin for evaluation the expression levels of cyclin D1 (*n* = 3). ^*^P < 0.05 vs. siNC group; ^#^*P* < 0.05 vs. Ctrl group.

Involvement of Gli-1 in regulation of LN229 cell growth was further investigated. Expression of Gli-1 was knocked down using a small interfering RNA technique. Western blot analysis showed that Gli-1 expression in LN229 cells was significantly blocked by transfection with Gli-1-targeted siRNA (siGli-1-1 and siGli-1-2), compared with that following siNC transfection. A high expression level of Gli-1 was found in a HepG2 cell line, which was used as a positive control in the experiments (Figures 6A,B). MTT assay demonstrated that Gli-1 downregulation significantly reduced LN229 cell viability. However, in the presence of the shh protein (shh, 5 μg/mL), one of the Hedgehog ligands that can increase the intracellular level of Gli-1, the viability of LN229 cells was remarkably increased (Figure 6C). Furthermore, the expression of cyclin D1 was suppressed in the Gli-1-targeted siRNA-transfected cells compared with that in siNC-transfected cells. Treatment with shh protein increased cyclin D1 expression (Figures 6D,E).

**Figure 7:**
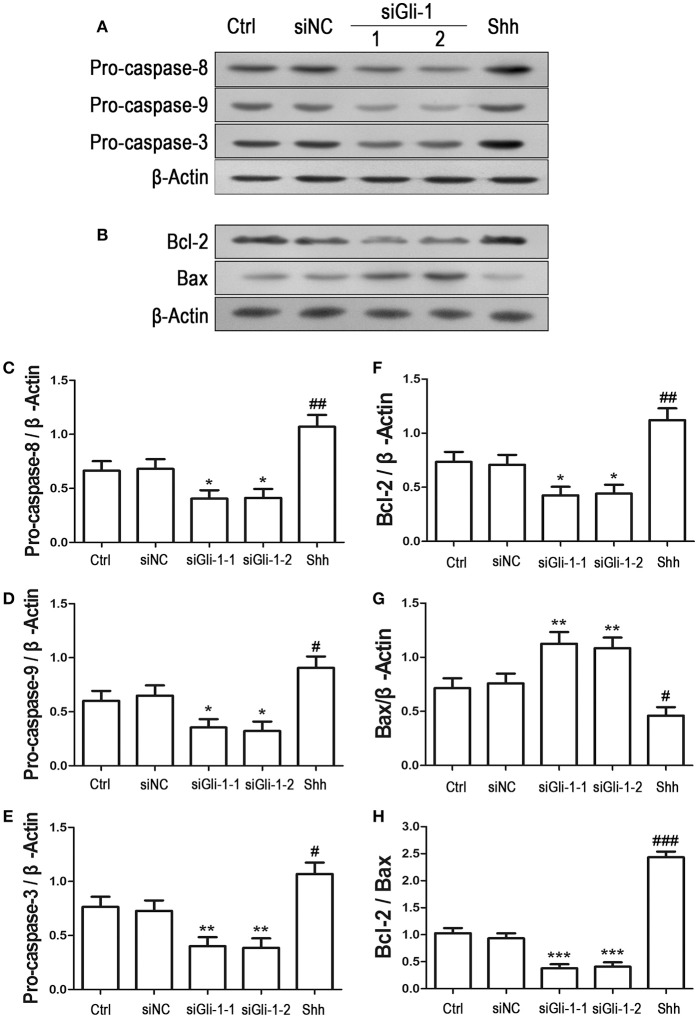
Effects of Gli-1 downregulation on the expression of apoptosis-related proteins in LN229 cells. **(A,B)** Cultured LN229 cells were transfected with non-specific siRNA (siNC) and two Gli-1-specific siRNAs (siGli-1-1 and siGli-1-2) for 24 h. Then, the expression of pro-caspase-8/9/3 **(A)** and Bcl-2/Bax **(B)** was determined by western blot (WB) analysis. Shh protein (5 μg/mL) was simultaneously added to the non-transfected LN229 cells as a positive control. **(C–H)** WB bands were quantified to generate the ratios of pro-caspase-8 **(C)**,−9 **(D)**, and−3 **(E)**, Bcl-2 **(F)**, and Bax **(G)** to β-actin and the ratio of Bcl-2 to Bax **(H)**; each statistical value represents the mean ± SD of three independent experiments (*n* = 5). ^*^P < 0.05, ^**^P < 0.01, ^***^P < 0.001 vs. siNC group; ^#^*P* < 0.05, ^##^*P* < 0.01, ^###^*P* < 0.001 vs. Ctrl group.

To examine the effect of Gli-1 downregulation on cell apoptosis, expression levels of the aforementioned apoptosis-related proteins were estimated in Gli-1-downregulated LN229 cells. The results revealed that the expression of pro-caspase-8/9/3 was reduced in Gli-1-targeted siRNA-transfected cells. Gli-1 knockdown also led to decrease of Bcl-2 level and increase of Bax level, which caused a decline in the Bcl-2/Bax ratio. Conversely, shh protein administration significantly increased pro-caspase-8/9/3 expression and the Bcl-2/Bax expression ratio (Figure 7). These findings were consistent with a recent investigation of medulloblastoma cells (Lin et al., 2016) and, implied an important role of Gli-1 in regulation of apoptosis and cell cycle progression in LN229 cells.

### Involvement of Gli-1 signal in mGluR4-mediated proliferation inhibition and apoptosis of LN229 cells

**Figure 8:**
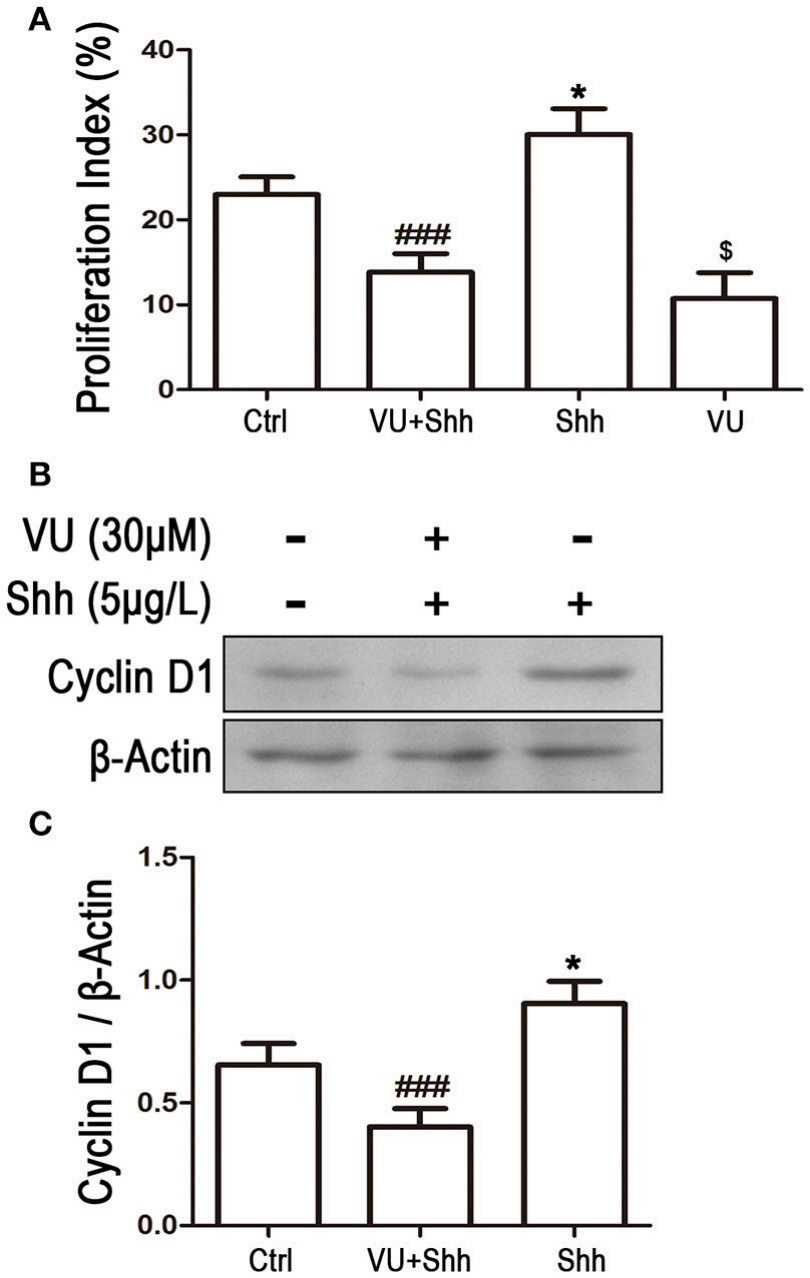
Involvement of Gli-1 in mGluR4-mediated cell cycle arrest of LN229 cells. LN229 cells were treated with the vehicle (Ctrl), 30 μM VU0155041, 5 μg/mL shh or 30 μM VU0155041 plus 5 μg/mL shh (VU + shh) for 24 h. **(A)** Cell cycle was detected by fluorescence activated cell sorting (FACS), and the result was indicated by Proliferation Index (PI), in which PI = [(S+G2/M)/(G0/G1+S+G2/M)]× 100%. ^*^,^$^*P* < 0.05 vs. Ctrl; ^###^*P* < 0.001 vs. shh group. **(B)** Representative western blot (WB) image illustrates the change of cyclin D1 expression. **(C)** WB bands were quantified to generate the ratio of cyclin D1 to β-actin. Each value represents the mean ± SD of three independent experiments. ^*^P < 0.05 vs. Ctrl; ^###^*P* < 0.001 vs. shh group.

To further identify whether the reduction in Gli-1 expression is involved in the growth inhibition and apoptosis of LN229 cells induced by mGluR4 activation, the cells were treated with 5 μg/mL of shh protein, which subsequently increased the expression level of Gli-1 and growth of the tumor cells. Then, 30 μM of VU was used to eliminate the effect of shh on Gli-1 expression and growth of LN229 cells. Cell proliferation and apoptosis were then assessed. Cell cycle analysis revealed that the percentage of proliferating cells (S + G_2_/M) was significantly increased by shh treatment as compared with that of the control, while VU treatment remarkably abolished the effect of shh on cell proliferation. VU in the absence of shh also played an inhibitory role in cell proliferation (Figure 8A). Similarly, the effect of shh on increasing expression of cyclin D1 was also attenuated by VU treatment (Figures 8B,C).

**Figure 9:**
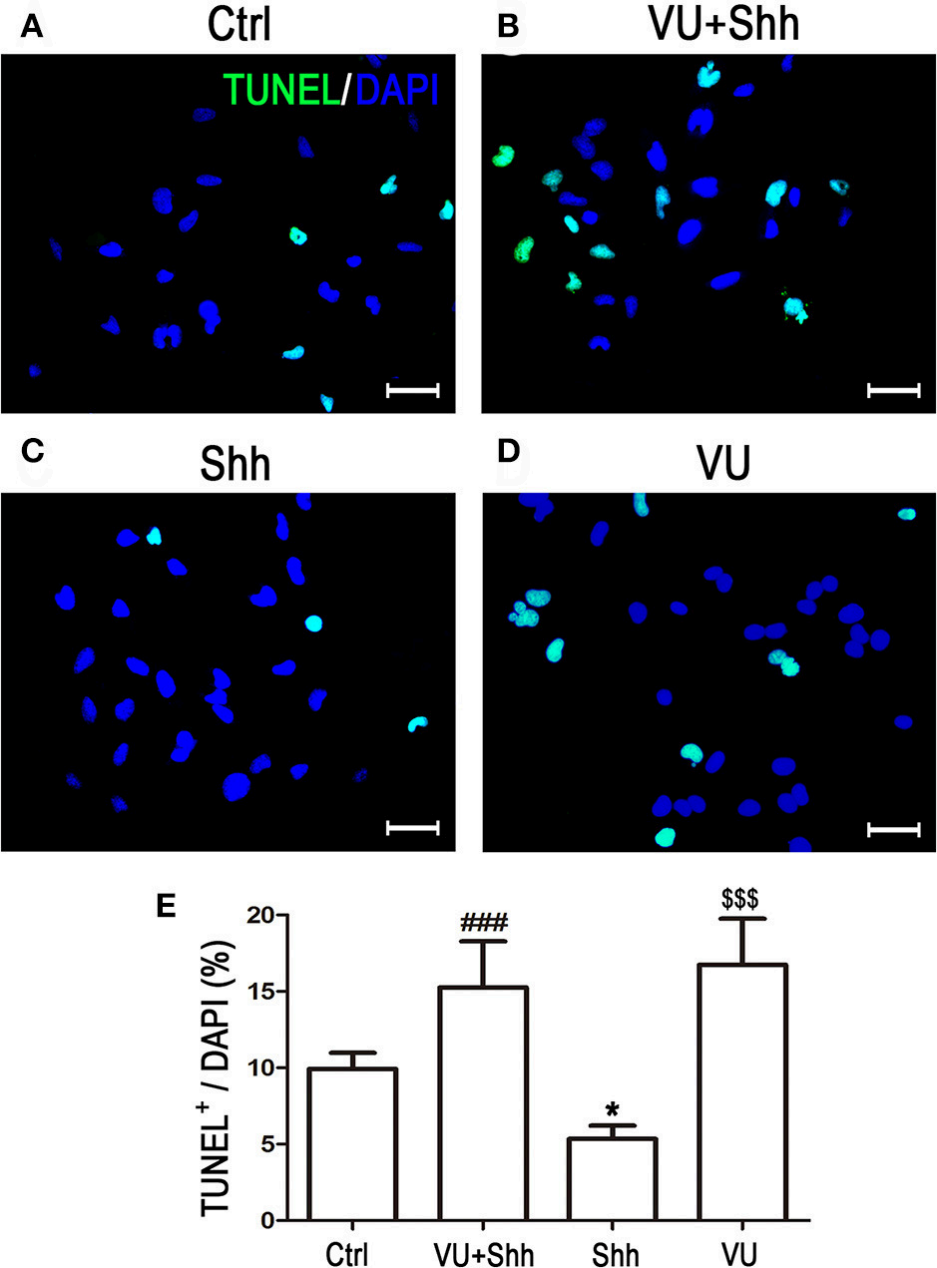
Involvement of Gli-1 inhibition in mGluR4-mediated death of LN229 cells. **(A–D)** LN229 cells were treated with the vehicle (Ctrl), 30 μM VU0155041, 5 μg/mL shh, or 30 μM VU0155041 plus 5 μg/mL shh (VU + shh) for 24 h. To estimate cell death, terminal-deoxynucleotidyl transferase-mediated dUTP nick end labeling (TUNEL, green) staining was performed after each treatment, and nuclei were counter-stained with DAPI (blue). Representative images are presented in **(A–C)**. Scale bar = 100 μm. **(E)** Quantitative analysis of TUNEL staining is shown, and data from at least three independent experiments are presented as the percentage of TUNEL positive cells to total DAPI-stained cells. ^*^P < 0.05, ^$$$^*P* < 0.001 vs. Ctrl; ^###^*P* < 0.001 vs. shh group.

**Figure 10:**
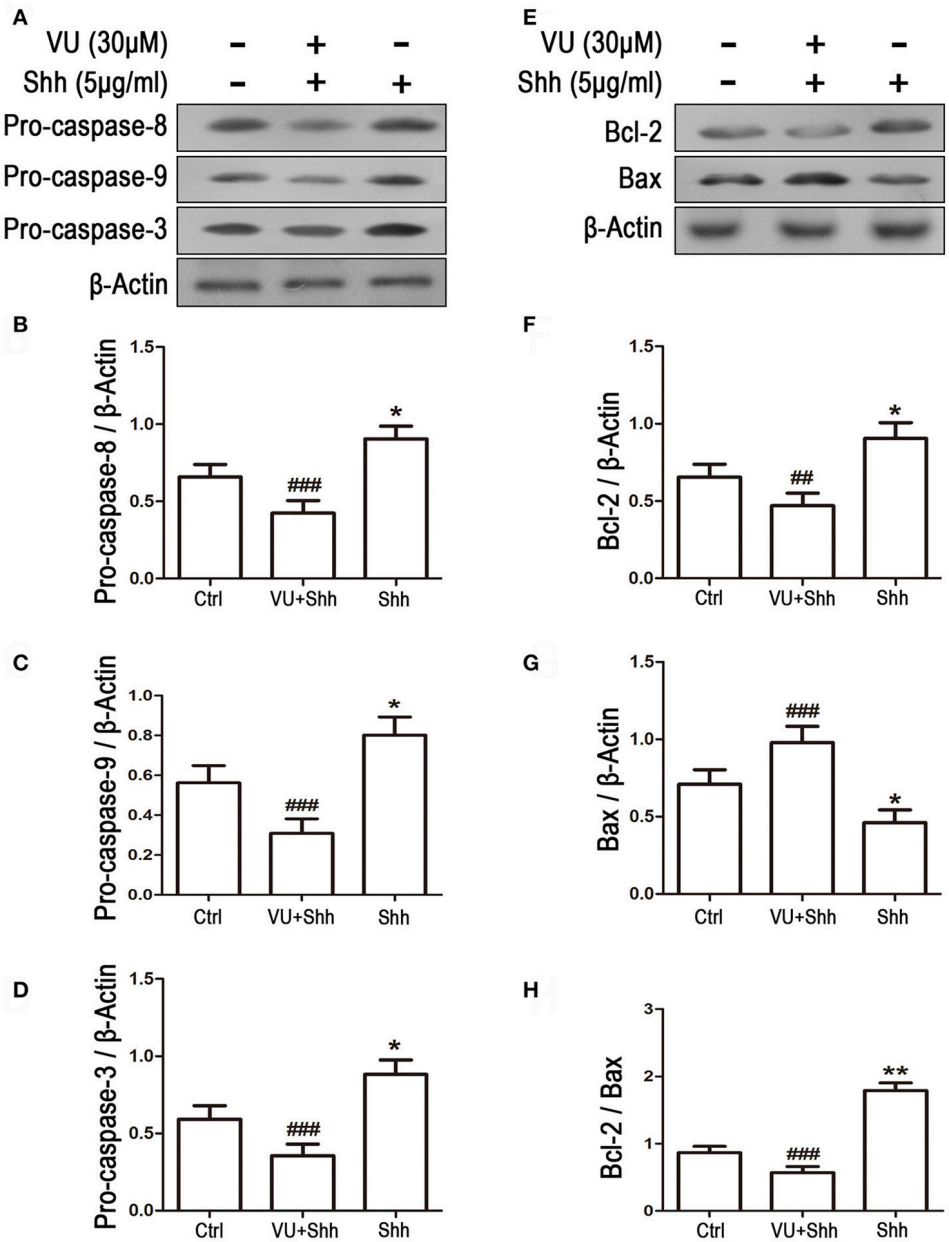
Gli-1 downregulation is involved in mGluR4-mediated dynamic expression of apoptosis-related proteins in LN229 cells. **(A,B)** LN229 cells were treated with the vehicle (Ctrl), 5 μg/mL shh, or 30 μM VU0155041 plus 5 μg/mL shh (VU + shh) for 24 h. Then, the expression of pro-caspase-8/9/3 **(A)** and Bcl-2/Bax **(B)** was determined by western blot (WB) analysis. **(C–H)** WB bands were quantified to generate the ratios of pro-caspase-8 **(C)**,−9 **(D)**, and−3 **(E)**, Bcl-2 **(F)**, and Bax **(G)** to β-actin and the ratio of Bcl-2 to Bax **(H)**; each statistical value represents the mean ± SD of three independent experiments. ^*^P < 0.05, ^**^P < 0.01 vs. Ctrl group; ^##^*P* < 0.01, ^###^*P* < 0.001 vs. shh group.

Cell apoptosis was assessed using TUNEL staining. As shown in Figure 9, shh treatment significantly decreased the number of TUNEL-positive cells, and VU treatment eliminated the inhibitory effect of shh on LN229 cell apoptosis, which enhanced cell apoptosis, compared with that of shh-treated cells. Consistently, VU treatment without shh remarkably increased apoptosis of LN229 cells. Correspondingly, treatment with shh substantially suppressed cleaved activation of pro-caspase-8/9/3, decreased the Bax level, and increased the Bcl-2 level and Bcl-2/Bax ratio, which imply a decrease of cell apoptosis. However, the regulatory role of shh in the expression of these apoptosis-related proteins was antagonized by VU administration (Figure 10), which suggests the potential involvement of Gli-1 downregulation in mGluR4-mediated inhibition of proliferation and enhancement of apoptosis of LN229 GBM cells.

## Discussion

In the present study, we demonstrated that LN229 cells, a GBM cell line, expressed mGluR4, and pharmacological activation of the receptor inhibited proliferation of the tumor cells, indicated by cell viability reduction, cell cycle arrest and downregulation of cyclin D1 expression. The tumor cells were also susceptible to apoptosis under VU treatment, which was revealed by activation of caspase-8/9/3 via cleaving and disruption of the Bcl-2/Bax expression balance. Moreover, mGluR4 activation negatively regulated the expression of Gli-1, whose activity plays essential roles in controlling the growth of glioma cells (Clement et al., 2007; Liu et al., 2014). The results suggested a possible role of mGluR4 in suppression of GBM growth, which may involve the inhibition of Gli-1 signaling in tumor cells.

Most research on the mGluRs was based on the agonist and antagonist methods and “synaptically oriented,” and these receptor subtypes have been considered as potential drug targets for neurological and psychiatric diseases (Bruno et al., 2000; Conn et al., 2005; Swanson et al., 2005; Nicoletti et al., 2007; Moyanova et al., 2011). However, recent findings showed that these receptors were expressed by and involved in the fundamental processes of developing cells, such as neural stem/progenitor cells and embryonic stem cells (Melchiorri et al., 2007; Zhao et al., 2011; Zhang et al., 2016). Interestingly, mGluRs have also been detected in non-neuronal cells that do not receive synaptic input, such as keratinocytes, hepatocytes, thymocytes, and osteoblasts (Skerry and Genever, 2001). Furthermore, subtypes of mGluRs have been found in several malignant tumor cells. mGluR1 has been identified in mouse melanoma cells and human melanoma biopsies, and its activation might support tumor growth through stimulating the MAP kinase signaling pathways (Pollock et al., 2003; Marín et al., 2006). mGluR3 is consistently expressed in human glioblastomas or glioma cell lines, and its antagonist LY341493 reduces cell growth with the inhibition of the MAP kinase and PI3K signaling pathways (D'Onofrio et al., 2003; Arcella et al., 2005). These findings might provide a novel therapeutic strategy for malignant cancers, in which mGluRs can be exploited as new targets for tumor treatment (Nicoletti et al., 2007).

Beyond expression in mature neurons for regulation of synaptic transmission and plasticity, mGluR4 is expressed in developing cells, including neural stem/progenitor cells and embryonic stem cells. Selective agonists of the receptor inhibit the proliferation and increase survival and neuronal differentiation of these cells (Canudas et al., 2004; Cappuccio et al., 2006; Saxe et al., 2007; Spampinato et al., 2014; Zhang et al., 2015). Moreover, mGluR4 is found in medulloblastomas, colorectal carcinomas and osteosarcomas (Yoo et al., 2004; Chang et al., 2005; Iacovelli et al., 2006; Jiang et al., 2014; Yang et al., 2014; Wang et al., 2016). Medulloblastomas, the most frequent malignant CNS cancers in children, originate from the transformed cerebellar granule cell progenitors. It has been found that mGluR4 expression is inversely associated with severity, spreading, and recurrence of the tumor. Moreover, mGluR4 enhancer PHCCC negatively regulated the growth of three medulloblastoma cell lines (Iacovelli et al., 2006), which implicated a novel potential drug target for glioma treatment. This prompted us to investigate the role of mGluR4 in glioblastoma, the most frequent glioma variant in the adult brain and among the deadliest of human malignant tumors (Sanai et al., 2005). As expected, mGluR4 was abundantly expressed in cultured LN229 glioblastoma cells. Furthermore, mGluR4 agonist VU reduced the viability and proliferation of LN229 cells and resulted in the increase of cell apoptosis. Compared to previous reports, these data may further support the role of mGluR4 in control of GBM growth.

In our study, the compound VU was used to activate specifically mGluR4 for observation of GBM cell proliferation and apoptosis. VU is a novel activator of mGluR4 that binds to the allosteric site of mGluR4. In addition to positively cooperating with orthsteric agonists (glutamate or L-AP4), another outstanding pharmacological characteristic of the activator is its significant intrinsic agonist activity, which was not attenuated by antagonist LY341495 (Niswender et al., 2008; Rovira et al., 2015). Furthermore, VU plays a specific role in activating mGluR4 without potentiating or abating other mGluR subtypes. Hence, VU may represent a breakthrough drug as well as a tool for the study of the role of mGluR4 in regulation of cell function in normal CNS and other cell types (Niswender et al., 2008; Betts et al., 2012; Guimarães-Souza and Calaza, 2012; Bogenpohl et al., 2013). In spite of its EC50 of 798 nM found in a human mGluR4 heterogeneous expression system, previous investigation reported that the effective concentrations of VU in cultured mouse cortical neurons were 10 and 30 μM, and VU did not exhibit obvious binding activity with off-target mGluR subtypes (Domin et al., 2016). Our previous study of cultured rat neural stem cells also demonstrated that the effective concentration of the compound was 30 μM in terms of regulation of cell proliferation and survival (Zhang et al., 2015). The present investigation showed that the effective concentrations were as high as 30 and 50 μM in terms of regulating the growth and down-stream intracellular signaling of the LN229 GBM cell line. The difference in effective concentration of VU may due to the different cell types observed, discrepancies in relative mGluR4 expression, and different pathophysiological states of cells. In the present experiment, we could not explore the possible off-target effects of VU under these concentrations in GBM cells, because we did not know the expression profiles of other mGluR subtypes in the cell line. Moreover, we did not confirm the desensitizing effect on mGluR4 during chronic exposure to VU and the possible involvement of PKC activation. Based on the present findings, future intensive investigations should be carried out in overexpression/knockdown models to resolve these key problems and further understand the possible application of VU in treatment of GBM cancers.

Until now, the underlying mechanisms of mGluR4 in regulation of glioblastoma remained unclear. It is interesting to note that mGluR4 enhancer PHCCC can decrease the level of Gli-1 in rat cerebellar granule cell precursors, while activation of the receptor inhibited the proliferation of the cells (Canudas et al., 2004). Moreover, it is well documented that Gli-1 mediates the mitogen effects of the SHH pathway in the regulation of growth of proliferating normal cells as well as several cancer cells (Dahmane et al., 1997, 2001; Briscoe and Therond, 2013). This inspired us to explore the effect of mGluR4 on Gli-1 expression in LN229 cells. The data displayed a significant reduction in Gli-1 expression induced by pharmacological activation of mGluR4, while gene-targeted elimination of mGluR4 rescued the Gli-1 reduction. These results may suggest a potential interaction between mGluR4 and Gli-1 signaling in regulation of GBM cell growth.

The SHH pathway regulates cell proliferation by promoting DNA replication and cell cycle entry through activity of three zinc-finger transcription factors (Gli-1, Gli-2, and Gli-3) (Katoh and Katoh, 2009). Gli-1 is involved in cell-fate determination, proliferation, and normal development patterning (Ruiz i Altaba, 1997). However, abnormal expression of Gli-1 is highly correlated with tumor development, such as in the brain, muscle, and skin (Dahmane et al., 1997; Nilsson et al., 2000; Shahi et al., 2012). Furthermore, dysregulation of Gli-1 may result in aberrant activation of the SHH pathway during initiation of brain glioma (Sanai et al., 2005). Consistently, our investigation showed that downregulation of Gli-1 substantially suppressed the mitosis of LN229 cells and consequentially increased cell apoptosis, suggesting a potential role of Gli-1 downregulation in the control of GBM tumor growth (Wang et al., 2010).

The SHH pathway tightly interacts with other signal pathways in regulation of the activity of normal and cancer cells (Riobó et al., 2006; Xia et al., 2012; Briscoe and Therond, 2013). For example, RTK signaling can potentiate the SHH pathway through PI3K/AKT-mediated GSK-3β suppression or RAS/STIL1-mediated SUFU inhibition (Bhatia et al., 2006; Yue et al., 2009). Interestingly, G-protein-coupled receptor signaling via G_α*i*_ can regulate SHH pathway activation through decreased intracellular cAMP concentration (Ogden et al., 2008). In our study, mGluR4, a typical G-protein-coupled receptor whose activation is negatively coupled to adenylate cyclase and reduction in intracellular cAMP level, was shown to decrease the Gli-1 level in LN229 cells, indicating that activation of mGluR4 might control the excessive expression of Gli-1, consequently inhibiting the growth of glioma cells through inactivation of the SHH pathway. Moreover, previous investigations indicated that activation of mGluR4 reduces the level of phosphorylated AKT in cerebellar granule cell progenitors (Canudas et al., 2004) and two medulloblastoma cell lines (Iacovelli et al., 2006). Unfortunately, we could not clarify the underlying mechanisms of mGluR4 regulation of the SHH pathway and the possible involvement of the AKT pathway in the control of LN229 cell growth of. Further investigations should be conducted to explore how mGluR4 activation influence the intracellular Gli-1 level and possible crosstalk between the SHH and PI3K/AKT pathways, which may be involved in the inhibitory action of mGluR4 on GBM cell growth.

The adult CNS harbors neural stem/progenitor cells, which can self-renew, proliferate, and differentiate into neurons and neuroglia cells (Zhao et al., 2008). In certain circumstances, it is highly possible that these cells are converted into cancer stem cells, which produce malignant glioblastomas by escaping the mechanisms that manage cell mitosis and programmed differentiation (Sanai et al., 2005). Considering the variety of developmental signaling pathways that are crucial mediators of tumorigenesis, such as the RTK/RAS/PI3K, P53, and SHH/Gli pathways (Tanaka et al., 2013), dysregulation of stem cell signals due to genetic predisposition and epigenetic alteration leads to carcinogenesis (Ingham and McMahon, 2001). Both in the developing and adult CNS, Gli-1 is expressed by neural stem/progenitor cells, and plays a vital role in maintenance of germinal niches by facilitating the survival and proliferation of stem cell progeny or supporting the stem cell population (Faigle and Song, 2013). It is also notable that Gli is expressed in low-grade and high-grade gliomas as well (Dahmane et al., 2001). Administration of cyclopamine, an inhibitor of SHH signaling, suppresses the proliferation of several glioma cell lines *in vitro* (Siegelin et al., 2009). Abnormal activation of SHH signaling might predispose to occurrence of medulloblastoma by inducing the inhibition of the retinoblastoma tumor-suppressor gene and directly inducing mediators of cell mitosis such as the proto-oncogene N-myc, which amplification and overexpression can lead to tumorigenesis (Kenney et al., 2003; Oliver et al., 2003). Hence, the inhibitory effect of mGluR4 activation on the growth of LN229 GMB cells might be due to suppression of the abnormal expression of Gli-1, which may restrict the activity of glioma stem cells.

In conclusion, our study demonstrated that mGluR4 inhibits the growth of the GBM cell line LN229; activation of mGluR4 not only inhibited cell proliferation but also promoted cell apoptosis. Furthermore, activation of mGluR4 downregulated the expression of the Gli-1 transcription factor, which accompanied the growth suppression of the LN229 GBM cell line. Hence, Gli-1 may be one of the key intracellular mediators in mGluR4 control growth of GBM cells. Our study might implicate a novel role of mGluR4, which can be employed as a potential drug target for therapy of glioblastoma.

## Author contributions

ZZ and XZ contributed to the project design, investigation, experiment execution, data analysis and original draft writing. YaL, YiL, and XL contributed to the experiment execution, data analysis and original draft writing. CL and HL contributed to the supervision experiments, data analysis and manuscript writing. XC and YoL contributed to the project design, investigation, data analysis, manuscript writing and proofreading, funding acquisition, and project administration.

### Conflict of interest statement

The authors declare that the research was conducted in the absence of any commercial or financial relationships that could be construed as a potential conflict of interest. The reviewer KKS and handling Editor declared their shared affiliation.
