# Supplementary material for: Activity of Metabotropic Glutamate Receptor 4 Suppresses Proliferation and Promotes Apoptosis With Inhibition of Gli-1 in Human Glioblastoma Cells
Source: Front Neurosci. 2018 May 15;12:320. doi: 10.3389/fnins.2018.00320 (PMC5962807; doi:10.3389/fnins.2018.00320)
Supplement: Supplementary file 1 [file Presentation_1.PDF]

Fig. S1

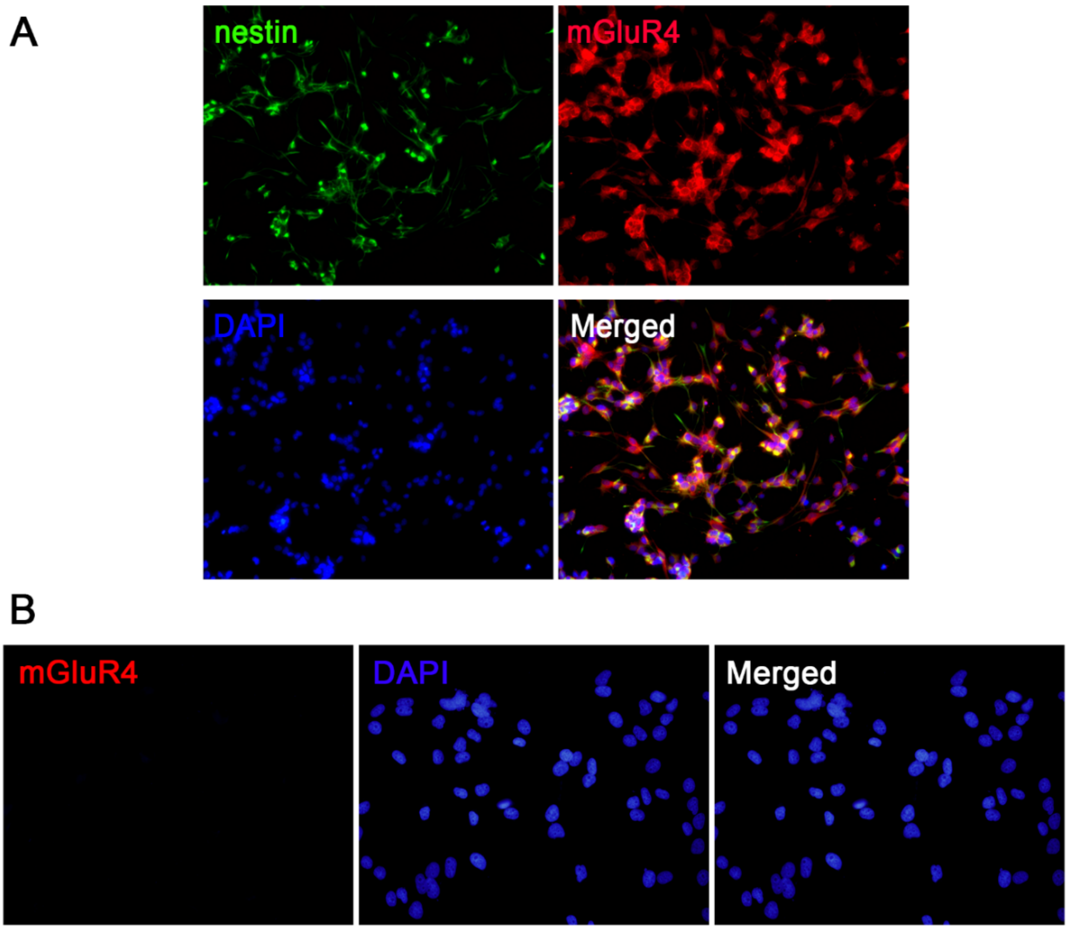

Fig. S1 Positive and Negative control for mGluR4 immunofluorescent staining. **(A)** the positive control was carried out in nestin (Green) positive cultured rat neural stem cells, which were reported by others and us that can express functional mGluR4 (red), and mGluR4 and nestin were demonstrated co-expression in the cells. (B) the negative control was performed by omitting the mGluR4 primary antibody in the staining procedure, which resulted in no positive red fluorescence detected in the samples. Nuclei in **(A)** and **(B)** were counter-stained with 4`,6-diamedino-2-phenylindole (DAPI, blue).

Fig. S2

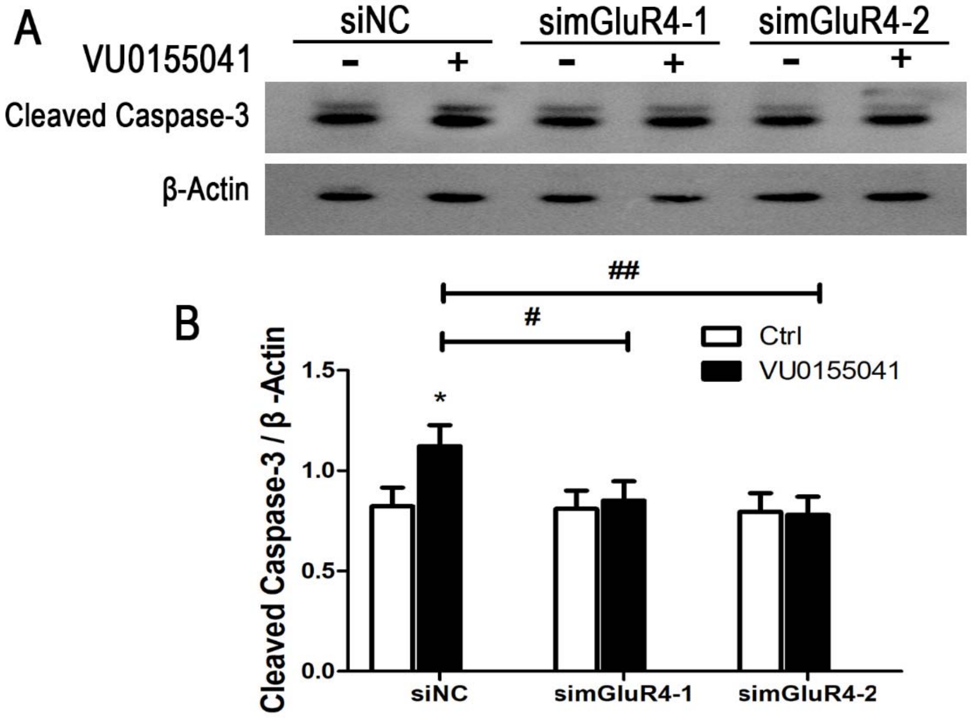

Fig. S2 **Effects of mGluR4 activation on the expression of Cleaved caspase-3 in U87MG GBM cell line. (A)** Cultured U87MG cells were transfected using non-specific siRNA (siNC) and two mGluR4-specific siRNA (simGluR4-1 and simGluR4-2) for 24 h, followed by treatment with the vehicle (Ctrl) or 30 μM of VU0155041 for 24 h. Then the differential expression of cleaved caspase-3 was determined by Western blot (WB) analysis. (B) WB band quantification for the ratio of P cleaved caspase-3 to β-Actin. *P<0.05 versus Ctrl group; #P<0.05, ##P<0.01 versus VU0155041 group.
